# A study of glycemic variability in patients with type 2 diabetes mellitus with obstructive sleep apnea syndrome using a continuous glucose monitoring system

**DOI:** 10.1186/s40842-020-00098-0

**Published:** 2020-06-05

**Authors:** Suhas S. Khaire, Jugal V. Gada, Ketaki V. Utpat, Nikita Shah, Premlata K. Varthakavi, Nikhil M. Bhagwat

**Affiliations:** 1 Department of Endocrinology, Room no. 419, 4th floor, College building, Topiwala National Medical College and Bai Yamunabai Laxman (B.Y.L.) Nair Charitable Hospital, A.L. Nair Road, Mumbai Central, Mumbai, Maharashtra 400008 India; 2 Department of Chest Medicine, Topiwala National Medical College and Bai Yamunabai Laxman (B.Y.L.) Nair Charitable Hospital, Mumbai, India

**Keywords:** Obstructive sleep apnea syndrome, Glycemic variability, Continuous glucose monitoring system, Diabetes mellitus with obstructive sleep apnea

## Abstract

**Background:**

Obstructive sleep apnea syndrome (OSAS) in association with Type 2 Diabetes Mellitus (DM) may result in increased glycemic variability affecting the glycemic control and hence increasing the risk of complications associated with diabetes. We decided to assess the Glycemic Variability (GV) in patients with type 2 diabetes with OSAS and in controls. We also correlated the respiratory disturbance indices with glycemic variability indices.

**Methods:**

After fulfilling the inclusion and exclusion criteria patients from the Endocrinology and Pulmonology clinics underwent modified Sleep Apnea Clinical Score (SACS) followed by polysomnography (PSG). Patients were then divided into 4 groups: Group A (DM with OSAS, *n* = 20), Group B (DM without OSAS, *n* = 20), Group C (Non DM with OSAS, *n* = 10) and Group D (Non DM without OSAS, *n* = 10). Patients in these groups were subjected to continuous glucose monitoring using the Medtronic iPro2 and repeat PSG. Parameters of GV: i.e. mean glucose, SD (standard Deviation), CV (Coefficient of Variation), Night SD, Night CV, MAGE and NMAGE were calculated using the Easy GV software. GV parameters and the respiratory indices were correlated statistically. Quantitative data was expressed as mean, standard deviation and median. The comparison of GV indices between different groups was performed by one-way analysis of variance (ANOVA) or Kruskal Wallis (for data that failed normality). Correlation analysis of AHI with GV parameters was done by Pearson correlation.

**Results:**

All the four groups were adequately matched for age, sex, Body Mass Index (BMI), waist circumference (WC) and blood pressure (BP). We found that the GV parameters Night CV, MAGE and NMAGE were significantly higher in Group A as compared to Group B (p values < 0.05). Similarly Night CV, MAGE and NMAGE were also significantly higher in Group C as compared to Group D (*p* value < 0.05). Apnea-hypopnea index (AHI) correlated positively with Glucose SD, MAGE and NMAGE in both diabetes (Group A plus Group B) and non- diabetes groups (Group C plus Group D).

**Conclusions:**

OSAS has a significant impact on the glycemic variability irrespective of glycemic status. AHI has moderate positive correlation with the glycemic variability.

## Background

Obstructive sleep apnea syndrome (OSAS) is characterized by repetitive episodes of cessation of breathing (apnea) or partial upper airway obstruction (hypopneas) during sleep, frequently associated with reduced blood oxygen saturation [1]. The prevalence of OSAS in patients with type 2 Diabetes Mellitus (DM) has been reported as 18 to 86% in the literature [2–4]. This association may impact glycemic control and worsen diabetes related complications [4]. OSAS is closely related to cardiovascular complications like coronary heart disease, heart failure, arrhythmias and sudden death at night [5–7], and is a risk factor for stroke and death after stroke [8]. Experimental studies have demonstrated that OSAS exerts adverse effects on glucose metabolism, such as worsening of insulin resistance, glucose intolerance and pancreatic β-cell dysfunction through complex neurohormonal mechanisms [9–13]. This association between OSAS and type 2 DM may be bidirectional as higher Glycated hemoglobin (HbA1c) levels were found even in non-diabetic patients with severe OSAS [14, 15]. Besides, OSAS is known to cause rapid fluctuations in blood glucose levels [15]. This Glycemic Variability (GV) is an independent risk factor for diabetes related complications, including cardiovascular diseases [16–18]. Therefore, GV may represent an important aspect of glycemia which is not reflected by conventional measures of glucose control, such as HbA1c and fasting plasma glucose [19]. Thus Continuous Glucose Monitoring System (CGMS) becomes a useful tool to assess GV in patients with DM. The data on the effect of OSAS on GV in type 2 DM is scarce. Hence we assessed the GV in patients with type 2 diabetes and OSAS by using a CGMS.

## Methods

### Aims

To assess the Glycemic Variability in patients with type 2 diabetes and OSAS. The primary objective was to correlate GV to respiratory disturbance indices in patients of type2 Diabetes with OSAS. The secondary objective was to assess GV in patients with OSAS without DM.

### Design and setting

This cross sectional study was conducted by the Endocrinology and Pulmonology services of a tertiary care center in Mumbai, Maharashtra, India, after Institutional Ethics Committee approval (No. ECARP/2018/95). We screened 55 patients with type 2 DM treated with lifestyle measures (LSM) alone or LSM with one or more oral antidiabetic medications (OAD). Patients aged between 18 to 60 years who had a BMI between 23 to 30 kg/m^2^ were eligible to participate in the study. The major exclusion criteria were patients with type 2 diabetes who were on insulin, type 1 diabetes, upper airway surgery in the past, sinusitis, chronic respiratory diseases, heart, lung, liver and kidney disease, patients who were taking sedative-hypnotic medications, pregnant patients, patients with history of alcohol consumption, smoking, steroid intake and untreated hypothyroidism. After applying inclusion and exclusion criteria, 40 patients with type 2 DM underwent further study. We included 10 patients with OSAS without diabetes from Pulmonology clinic and 10 were normal volunteers without diabetes. After a written informed consent participant’s baseline characteristics, anthropometric and clinical data were collected by a single investigator.

Patients were screened for OSAS with the help of Modified Sleep Apnea Clinical Score (SACS) which is a pre-test probability score- based on snoring (3 points), witnessed episodes of apnea (3 points), neck circumference (in cm) and systemic hypertension (4 points). Based on SACS patients were categorized into low (below 43), medium (43–48) and high risk (above 48) Groups [20, 21]. SACS score was used as a screening tool to identify patients with high probability of OSAS and to avoid unnecessary screening polysomnography (PSG) in a resource limited setting. Patients in moderate and severe groups were subjected to a screening polysomnography (PSG) for confirmation of the diagnosis. Patients in the low risk group willing to participate in the study were also screened with PSG with an intent to classify them into control groups. PSG was done with RESMED’s Apnea link device in the sleep laboratory of the Pulmonology department. This was a limited 5 channel level 3 PSG involving measurement of cardiovascular variables. It measured snoring, respiratory effort, pulse, oxygen saturation and nasal flow. The subjects were monitored for sleep apnea starting at 22:00 h. The sleep monitoring equipment was worn for at least 7 h and was removed by the specialist the next morning after the patient awoke. During the test, the patients did not have access to sedatives, coffee and tea. The Apnea Hypopnea Index (AHI) was calculated based on the total number of sleep apneas and hypopneas per hour. OSAS was defined as per American association of sleep medicine (AASM) criteria [22]. Patients with AHI ≥5 /hour, were classified as having obstructive sleep apnea. Four patients with low SACS score had significant AHI scores and were included in the obstructive sleep apnea group. The patients were categorized into 4 groups based on AHI:

Group A: DM with OSAS (*n* = 20 patients),

Group B: DM without OSAS (*n* = 20 patients),

Group C: Non DM with OSAS (*n* = 10 patients),

Group D: Non DM without OSAS (*n* = 10 patients).

On day 1, a fasting blood sample was drawn after a 12 h overnight fast between 8 to 9 am for routine biochemistry and HbA1c. HbA1c was determined by High Performance Liquid Chromatography using BioRad D10 Analyzer (Intra and Inter assay coefficient of variation- < 2%).

**Fig. 1:**
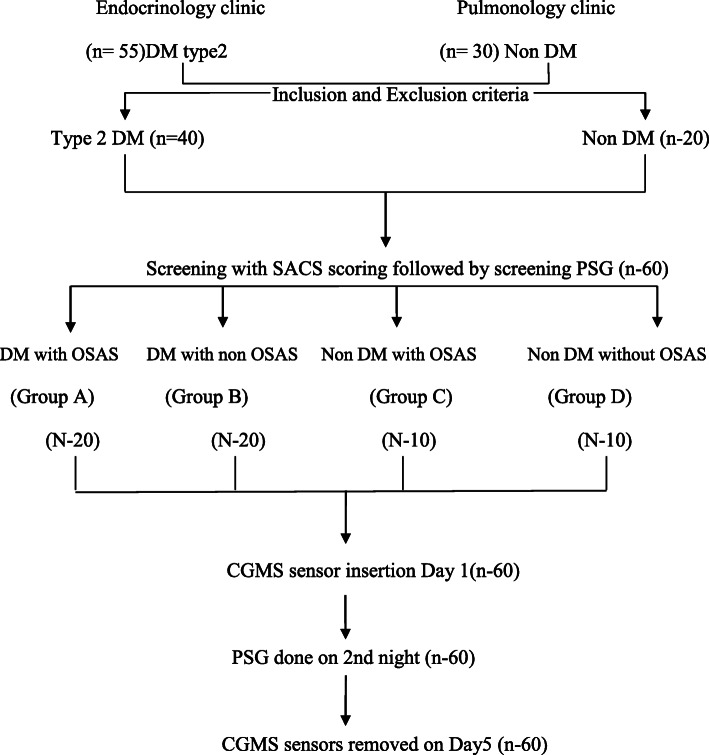
Flowchart of Methodology

Continuous glucose monitoring with the iPro2 CGM (Model REF-MMT 7102 W, Medtronic MiniMed, USA) was initiated for 5 days. The sensor was inserted subcutaneously over the anterior abdominal wall. The instrument was calibrated by four capillary glucose values obtained on a glucometer (Freestyle Optium Xceed) prior to three major meals and at bedtime. Blood glucose meters and the test strips were provided to the patient. Patients were instructed to be consistent with their meal timings, pattern and maintain a food diary which was analyzed by a registered dietitian. The patients were subjected to a second PSG on the second night of CGMS insertion. Fig. 1.

CGM data was downloaded with the CareLink Ipro1 software (MMT-7340) (Medtronic, Minneapolis, MN, USA) and this data was used to calculate the variability parameters by an automated Software EasyGV version 9.0.R2. Glycemic variability was defined as intraday glycaemic excursions, including episodes of hyperglycemia and hypoglycemia. Following variables were calculated from CGM readings for each patient: Time In Range (TIR), Time Above Range (TAR) and Time Below Range (TBR), Mean glucose, Standard deviation (SD), Night SD, Coefficient of Variation (CV), Night CV, Mean Amplitude of Glycemic excursion (MAGE) [23] and Night mean amplitude of glycemic excursion (NMAGE). NMAGE was calculated as MAGE for the night period and was recorded from 10 pm to 6 am.

Sample size was calculated based on the study of glycaemic variability and OSA by Nakata K, et al [24]; at 80% power and 5% alpha error by comparison of the mean method, the minimum sample size was found to be 18 per group in diabetes cohort.

Statistical analysis was done using the SPSS v 20.Demographic data was analyzed using descriptive statistics. Statistical significance was set to *P* <  0.05. Quantitative Data was expressed as mean, standard deviation and median. The comparison of GV indices between different groups was performed by one-way analysis of variance (ANOVA) for normally distributed data. For data parameters which failed the normality test, Kruskal Wallis test was applied. Post hoc analysis for multiple intergroup comparisons was done using Tukey HSD after ANOVA or Dunn’s method after Kruskal Wallis test. Correlation analysis of AHI with SD/ MAGE/NMAGE was done by Pearson correlation.

## Results

### Demographic data

We had 20 patients each in group A and group B and10 patients in group C and group D. The four study groups were adequately matched for age, sex, BMI, waist circumference and blood pressure. Table 1 shows the baseline characteristics of the cohort.

**Table 1:** Baseline Characteristics of the Study Population

VARIABLE	Group A (*n* = 20) Mean ± SD	Group B(*n* = 20) Mean ± SD	Group C (*n* = 10) Mean ± SD	group D (*n* = 10) Mean ± SD	*p* value
Age (Years)	49.40 ± 7	46.25 ± 11.86	45.90 ± 11.32	44.80 ± 10.98	0.619
Sex(M:F)	15: 5	15: 5	7: 3	7: 3	0.902
BMI (kg/m2)	23.98 ± 2.15	23.63 ± 2.59	23.46 ± 2.19	24.15 ± 3.06	0.898
WC (cm)	91.45 ± 5.25	92.45 ± 5.74	91.40 ± 4.81	91.60 ± 5.48	0.931
SBP^**a**^(mm/Hg)	130.40 ± 10.75	125.60 ± 9.12	129.80 ± 9.35	129.20 ± 9.20	0.420
DBP (mm/Hg)	85.90 ± 6.88	82.90 ± 6.10	85.60 ± 5.64	84.20 ± 5.03	0.415
SACS score ^b^	45.10 ± 4.24	37.45 ± 4.39	45 ± 6.25	34.70 ± 7.39	**0.0001**
Hb1Ac^**a**^ (%)	8.87 ± 2.54	8.18 ± 2.16	5.19 ± 0.43	4.82 ± 0.3	0.08
FPG^**a**^(mmol/L)	7.32 ± 1.74	7.57 ± 2.29	5.10 ± 0.47	4.85 ± 0.20	0.07

*P* value indicates comparison between all four groups. *P* <  0.05 is considered significant.

One way ANOVA applied

^a^ Data failed normality hence Kruskal Wallis applied

^b^ Ordinal data hence Kruskal Wallis applied

Patients with Diabetes Mellitus who were on lifestyle modifications alone, or on Lifestyle modification with monotherapy or combination therapy with 2 or 3 oral antidiabetic drugs. Fifty percent patients had diabetes duration of more than 5 years. 47.5% (19 out of 40) had retinopathy and 60%(24 out of 40) had peripheral neuropathy **(**Table 2**).**

**Table 2:** Profiles of patients with diabetes

VARIABLE	Group A (***n*** = 20)	Group B(***n*** = 20)
**Medications(n)**		
Life style modification	1	3
Metformin	5	3
Metformin + Glimepiride	4	4
Metformin + DPP4 inhibitor	8	8
Metformin+ Glimepiride + DPP4 inhibitor	2	2
**Duration of DM**		
Less than 5 yrs.	12	8
More than 5 yrs.	8	12
**Complications(n)**		
Retinopathy (Non Proliferative)	9	10
Peripheral Neuropathy	12	12

HbA1c was comparable in Group A and B (8.87 ± 2.54% vs. 8.18 ± 2.16%) *P* value > 0.05. SACS was higher in the group A (45.10 ± 4.24) and was statistically significant (*p* <  0.00012) when compared with rest of the three groups. Four patients with low SACS had positive polysomnography. The BMI of our patients were in the category of overweight and obese as per the Indian definition of BMI [25]. This is well described as thin fat Indian phenotype [25].

CGMS parameters:

Mean glucose (7.77 ± 2.42 mmol/L), glucose SD (2.44 ± 1.10 mmol/L), Night CV (31% ± 4.5) MAGE (5.20 ± 2.15 mmol/L) and NMAGE (5.78 ± 2.16) were highest in Group A as compared to rest of the three groups which was statistically significant (p values 0.006, 0.008, 0.008, 0.01 respectively) **(**Table 3**).** Post hoc analysis was then done to compare group A with group B and group C with group D.

**Table 3:** Comparison of the Glycemic variability indices and AHI in the study population

VARIABLE	Group A (*n* = 20) Mean ± SD	Group B (*n* = 20) Mean ± SD	Group C (*n* = 10) Mean ± SD	Group D (*n* = 10) Mean ± SD
AHI	18.97 ± 13.59	3.50 ± 1.34	18.98 ± 13.72	3.51 ± 1.15
Time in Range (%)	67 ± 4.26	69 ± 3.54	89 ± 2.71	97 ± 1.33
Time above Range (%)	20 ± 2.5	19 ± 2.8	8 ± 1.2	0
Time below range (%)	13 ± 2.1	12 ± 1.8	3 ± 1.4	2 ± 0.5
Mean Glucose (mmol/L)	7.77 ± 2.42	6.81 ± 2.31	5.85 ± 2.10	4.83 ± 1.95
SD (mmol/L)	2.44 ± 1.10	1.91 ± 0.86	0.87 ± 0.16	0.68 ± 0.11
CV (%)	31% ± 4.5	28% ± 3.5	16% ± 2.8	14% ± 2.4
Night Mean glucose (mmol/L)	6.84 ± 1.82	6.70 ± 1.64	5.05 ± 0.20	4.70 ± 0.15
Night SD (mmol/L)	2.54 ± 1.76	1.96 ± 0.92	0.98 ± 0.14	0.74 ± 0.14
Night CV (%)	37 ± 2.5	29 ± 2.3	19 ± 1.2	15 ± 1.1
MAGE (mmol/L)	5.20 ± 2.15	2.67 ± 1.04	2.47 ± 0.42	0.77 ± 0.19
NMAGE (mmol/L)	5.78 ± 2.16	2.22 ± 1.00	2.85 ± 0.42	0.62 ± 0.15

One way ANOVA applied, data failed normality hence Kruskal Wallis test applied

*P* value was significant between all four groups

*P* <  0.05 was considered significant

Time in Range: 3.9 to 10 mmol/L, Time Below range: < 3.9 mmol/L, Time above range > 10 mmol/L

### Glycemic variability in group a and group B: (Table 4)

Mean glucose (7.77 ± 2.42 vs. 6.81 ± 2.31 mmol/L), glucose SD (2.44 ± 1.10 vs. 1.91 ± 0.86 mmol/L), MAGE (5.20 ± 2.15 vs. 2.67 ± 1.04 mmol/L) and NMAGE (5.78 ± 2.16 vs. 2.22 ± 1.00 mmol/L) were higher in Group A than Group B. Post hoc analysis with multiple comparisons (Dunn’s method) in subgroup analysis, showed MAGE and NMAGE were significantly different between group A and group B (*p* <  0.05). The measures of % CV and SD were not significant but the night CV was significantly different (*P* < 0.05).

**Table 4:** Glycemic variability indices and AHI (Group A & Group B)

VARIABLE	Group A (***n*** = 20) Mean ± SD	Group B (***n*** = 20) Mean ± SD	***p*** value
AHI	18.97 ± 13.59	3.50 ± 1.34	**< 0.05**
Mean Glucose (mmol/L)	7.77 ± 2.42	6.81 ± 2.31	> 0.05
SD (mmol/L)	2.44 ± 1.10	1.91 ± 0.86	> 0.05
CV (%)	31% ± 4.5	28% ± 3.5	> 0.05
Night Mean glucose (mmol/L)	6.84 ± 1.82	6.70 ± 1.64	> 0.05
Night SD (mmol/L)	2.54 ± 1.76	1.96 ± 0.92	> 0.05
Night CV (%)	37 ± 2.5	29 ± 2.3	**< 0.05**
MAGE (mmol/L)	5.20 ± 2.15	2.67 ± 1.04	**< 0.05**
NMAGE (mmol/L)	5.78 ± 2.16	2.22 ± 1.00	**< 0.05**

Post hoc analysis (Dunn’s method) in subgroups

*P* < 0.05 is significant

### Glycemic variability in group C and group D: (Table 5)

Comparing the Non Diabetes groups (group C and D), we found the mean glucose (5.85 ± 2.10 vs. 4.83 ± 1.95 mmol/L), glucose SD (0.87 ± 0.16 vs. 0.68 ± 0.11 mmol/L),MAGE (2.47 ± 0.42 vs. 0.77 ± 0.19 mmol/L) NMAGE (2.85 ± 0.42 vs. 0.62 ± 0.15 mmol/L) and Night CV (37 ± 2.5 vs. 29 ± 2.3) were higher in group C (with OSAS) as compared to those in group D. MAGE, NMAGE and Night CV were statistically significant (*p* value < 0.05). The TAR in group C was 8% ± 1.2 whereas it was 0% in group D.

**Table 5:** Glycemic variability indices and AHI in Group C and Group D

VARIABLE	Group C (***n*** = 10) Mean ± SD	Group D (***n*** = 10) Mean ± SD	***p*** value
AHI	18.98 ± 13.72	3.51 ± 1.15	**< 0.05**
Mean Glucose (mmol/L)	5.85 ± 2.10	4.83 ± 1.95	> 0.05
SD (mmol/L)	0.87 ± 0.16	0.68 ± 0.11	> 0.05
CV (%)	16% ± 2.8	14% ± 2.4	> 0.05
Night Mean glucose	5.05 ± 0.20	4.70 ± 0.15	> 0.05
Night SD	0.98 ± 0.14	0.74 ± 0.14	> 0.05
Night CV (%)	19 ± 1.2	15 ± 1.1	**< 0.05**
MAGE (mmol/L)	2.47 ± 0.42	0.77 ± 0.19	**< 0.05**
NMAGE (mmol/L)	2.85 ± 0.42	0.62 ± 0.15	**< 0.05**

Post hoc analysis between subgroups (Dunn’s method)

### Glycemic profile in group A and group B from 10 pm to 6 am

**Fig. 2:**
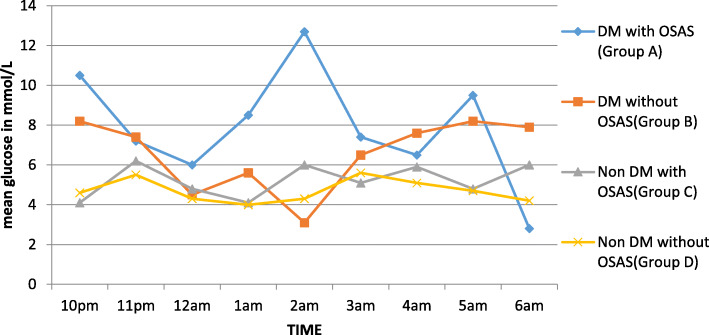
Mean glucose profile curves of all groups from 10 pm to 6 am

We compared the mean glucose profile curves from 10 pm to 6 am in group A and group B. There was a higher variability in group A as compared to group B. The mean peak glucose in group A and group B was 12.76 mmol/L and 11.65 mmol/L whereas the mean nadir glucose value was 2.83 mmol/L and 3.1 mmol/L respectively Fig. 2.

Patients showing increased glycaemic variability in group A than group B and increased variability in group C than group D.

### Glycemic profile in (group C and group D) from 10 pm to 6 am

We also compared the mean glucose profile curves from 10 pm to 6 am in group C and group D. Group C showed a higher variability as compared to group D. The mean peak glucose level of group C and group D was 6.2 mmol/L and 5.5 mmol/L whereas mean nadir glucose value was 4.1 mmol/L and 3.99 mmol/L respectively.

### Correlation of AHI with GV parameters in patients with Diabetes (Group A + Group B) Fig. 3a

AHI positively correlated with Glucose SD, MAGE and NMAGE with correlation coefficient of 0.219, 0.464, and 0.521 respectively in patients with diabetes (group A and B) with/without OSAS on Pearson’s correlation. This finding denotes moderate correlation of OSAS severity with glycemic variability indices.

**Fig. 3:**
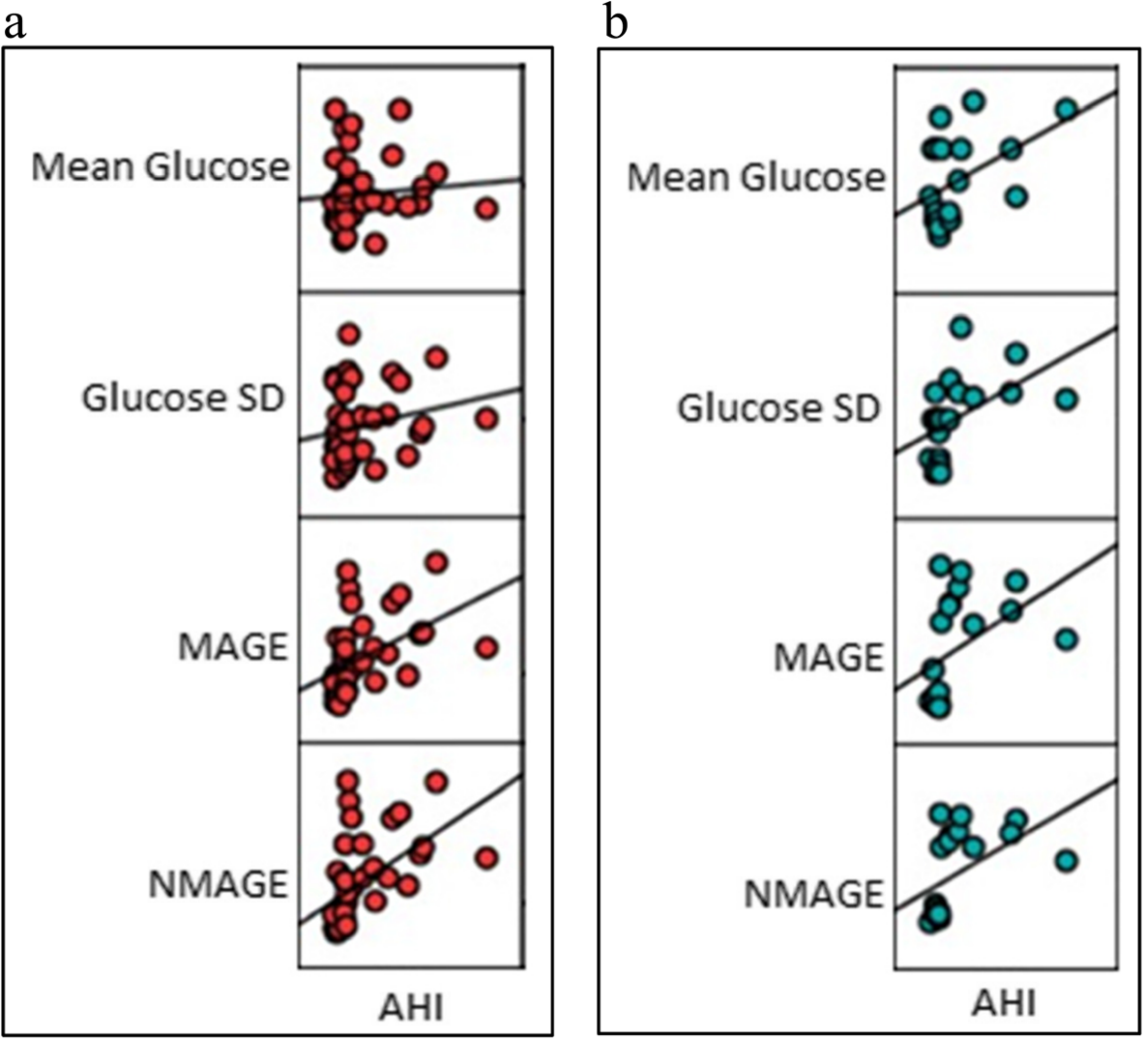
**a**: Pearson’s correlation between AHI and GV parameters in diabetes groups (OSAS + Non OSAS) (n-40). **b**: Pearson’s correlation between AHI and GV parameters in non-diabetes groups (OSAS + Non OSAS) (n-20)

### Correlation of AHI with GV parameters in patients without diabetes (Group C + Group D) Fig. 3b

AHI also correlated positively (moderate correlation) with glycemic variability parameters of Glucose SD, MAGE and NMAGE with correlation coefficient of 0.494, 0.438 and 0.495 respectively on Pearson’s correlation.

## Discussion

The association of sleep apnea with diabetes mellitus is bidirectional [14]. Most studies have reported the correlation of AHI with plasma glucose levels, HbA1c and insulin resistance indices [14, 15], however very few studies have looked at the glycemic variability parameters. Our study is probably the first to study GV in DM and non DM groups with and without OSAS. All the four groups studied were adequately matched for all the confounding factors such as age, sex, BMI, waist circumference and blood pressure which could affect OSAS.

We found that the MAGE, NMAGE and Night CV were significantly higher in group A than group B even though the TIR, TAR, TBR, CV and SD were not significantly different. This indicates that patients with similar levels of glycemia had higher glycemic variability especially at night suggesting a role of OSAS. Similarly Nakata et al.in his study found significantly higher MAGE and NMAGE in his group of patients who had diabetes and OSAS as compared to group OSAS without diabetes [24]. When we compared our Group A patients with their patients who had diabetes with OSAS, our patients had higher NMAGE (5.78 mmol/L vs. 2.75 mmol/L) but lower AHI (18.97 vs. 29) with a comparable MAGE (5.20 mmol/L vs. 5.30 mmol/L). The characteristics of study population in Nakata et al. was significantly different than our study population which could explain the differences in GV parameters. They had patients with heart failure (54.7%), patients on insulin and CKD which were exclusions for our study.

The parameters of Night CV, MAGE and NMAGE were significantly higher in group C compared to group D. These finding were similar to those with the study done by Peng et al. [26] who had recruited OSAS (n- 80) and non OSAS (n-40) patients but both without diabetes. Our patients in Group C (Non-diabetes with OSAS) had lower MAGE (2.47 mmol/L vs. 4.00 mmol/L) and comparable NMAGE (2.85 mmol/L vs. 1.99 mmol/L) with respect to their study group. This difference could be attributed to smaller sample size in our study, higher AHI and higher BMI in their cohort. The above findings shows that OSA has an impact on glycemic variability in patients without diabetes also.

Patients in group C (Non DM with OSAS) had at least 8% readings as TAR with significantly higher night CV compared to the group D (non DM with non OSA). This could be attributed to the OSAS component in group C.

The waist circumference and BMI of our population is characteristic of the thin fat Indian phenotype with centripetal obesity and higher insulin resistance [25]. Besides this, glycemic variability is a risk factor not only for endothelial damage but may also cause apoptosis of β cells [27]. It would be interesting to consider that patients in group C with higher TAR and GV would probably be at a higher risk of developing diabetes in future.

When AHI was correlated with the GV indices (Glucose SD, MAGE and NMAGE) in the diabetes and non-diabetes group we found a moderate positive correlation which implies that as the AHI increases there is greater increase in GV. However the study by Nakata et al. did not find any significant correlation in their DM group. The study by Peng et al. in non DM patients did find positive correlation of AHI with MAGE and NMAGE. With the results of our study it would be tempting to speculate that the benefit of treating OSAS would also be beneficial in reducing GV rather than just improving glycemic control. It would be worthwhile to see whether changes in glycemic variability reverse after application of Continuous Positive Airway Pressure therapy (CPAP) in OSAS with diabetes. OSAS is a known, albeit an underappreciated comorbidity of type 2 diabetes mellitus. It may be one of the contributors to poor glycemic control and should be actively sought at least in patients with symptoms suggestive of OSAS.

Limitation of the study was low sample size especially in the Non-diabetes groups, hence studies with larger sample size would help to better predict the relation between diabetes/non diabetes and OSAS.

## Conclusion

OSAS has a significant impact on Glycemic Variability in presence or even in absence of diabetes. Respiratory disturbances index (AHI) has moderate correlation to glycemic variability indices.

## Data Availability

Not applicable.

## Abbreviations

DM: Diabetes Mellitus
OSAS: Obstructive sleep apnea syndrome
GV: Glycemic variability
AHI: Apnea hypopnea index
SACS: Sleep Apnea Clinical Score
PSG: Polysomnography
CGMS: Continuous glucose monitoring system
MAGE: Mean amplitude of glycaemic excursions
NMAGE: Night mean amplitude of glycemic excursions
SD: Standard Deviation
CV: Coefficient of variation
TIR: Time in Range
TBR: Time below Range
TAR: Time above Range
LSM: Lifestyle Modifications
OAD: Oral Antidiabetic Medications
DPP4: Dipeptidyl Peptidase 4
BMI: Body Mass index
WC: Waist Circumference
SBP: Systolic Blood Pressure
DBP: Diastolic Blood Pressure
FPG: Fasting Plasma Glucose
